# The Influence of Social Determinants of Health on Treatment Delivery and Outcomes in Acute Leukemia

**DOI:** 10.1007/s11899-026-00792-3

**Published:** 2026-11-04

**Authors:** Jordan Atkins, Jerry Luo, Hae Lin Cho, Hannah Johnston, Danyal Shah, Stephanie Tsai, Mark Fiala, Andrew Hantel, Anand Patel, Irum Khan

**Affiliations:** 1 Division of Hematology/Oncology, and the Robert H Lurie Comprehensive Cancer Center, Northwestern University, 303 E Superior Ave, Ste 5-117, Chicago, IL 60611 USA; 2 Division of Pediatric Hematology, Oncology, Stem Cell Transplantation & Regenerative Medicine, Stanford University, Palto Alto, CA USA; 3 Department of Medical Oncology, Dana-Farber Cancer Institute, Boston, MA USA; 4 Section of Hematology/Oncology, Department of Medicine, University of Chicago, Chicago, IL USA; 5 College of Arts and Sciences, Washington University, St Louis, MO USA; 6 Division of Hematology/Oncology, Loyola University Medical Center, Chicago, IL USA; 7 Division of Oncology, Washington University, St Louis, MO USA; 8 Center for Bioethics, Harvard Medical School, Boston, MA USA

## Abstract

**Purpose of Review:**

Acute leukemia outcomes have conventionally been predicated on the genomic characteristics of leukemia cells. However, emerging evidence from population-based and national co-operative group studies suggests a pivotal role of social determinants of health (SDOH) on acute leukemia survival outcomes.

**Recent Findings:**

Acute leukemia outcomes over the past two decades have benefited from advances in molecular diagnostics, which have ushered in a multitude of targeted therapies including small molecule inhibitors and immunotherapies, liberalization of donor matching for transplantation, and early incorporation of supportive care. However, these advances have not benefited all populations equally. Environmental exposures, insurance status, access to transportation, and language barriers are some evaluable domains of social determinants of health (SDOH) that have been linked to leukemia outcomes. SDOH can influence leukemia outcomes through impact on patient access to specialized care, clinical trial enrollment, allogeneic stem cell transplantation, and palliative care. Beyond well-established disparities in access to care, emerging epidemiologic evidence suggests that SDOH may influence the genomic and biological characteristics of leukemia. Clinical prognostic leukemia models such as ELN 2022 do not accurately predict chemotherapy outcomes in diverse patient populations. This may be attributable to biologic differences in leukemia conferred by the social and heritable aspects of race. With the current landscape shifting to incorporate small-molecule inhibitors and immunotherapies into the frontline treatment of leukemia, SDOH-informed risk stratification can optimize prognostication and guide vulnerable patients toward less toxic, equally effective therapeutic alternatives.

**Summary:**

This review summarizes current evidence on how social determinants of health can influence multiple aspects of leukemia biology and outcomes and provides a framework to address the influence of SDOH along the continuum of acute leukemia care.

## Operationalizing Measures of Social Determinants of Health

Social Determinants of Health (SDOH) are the broader economic, political, and environmental systems that shape a person’s capacity to achieve optimal health outcomes [1]. These non- medical factors encompass the daily conditions in which individuals are born, grow, work, live, and age, and fundamentally reflect the unequal distribution of power, wealth, and systemic resources across populations. SDOH are a vital part of the clinical equation, shaping the trajectory of a disease in tandem with underlying biology and individual behaviors. Within modern health research, SDOH are generally categorized into five overarching domains:

(1) economic stability, (2) education access and quality, (3) health care access and quality, (4) neighborhood and built environment, and (5) social and community context [2]. These are complex, theoretical domains without “gold-standard” measures [3]. Moreover, they are largely absent from clinical databases and cancer registries, and available data are often limited to patient marital status, occupation, and type of health insurance (e.g., Medicaid/public, private, or uninsured) [4].

To overcome the absence of structured, individual-level SDOH data, many researchers operationalize SDOH through broad area-based measures that quantify the overall economic and social conditions of where a person lives, a concept referred to as neighborhood-level socioeconomic status (nSES) [2]. Measures of nSES can be single-dimensional, such as median household income, or composite scores, such as the Social Vulnerability Index (SVI) and Area Deprivation Index (ADI) [5]. While these area-level proxies have been statistically linked to variations in survival and treatment-related outcomes in acute leukemias at the population-level, they assume that all individuals within a geographic boundary share the same socioeconomic risks. This obscures differences within the larger community, and these measures frequently fail to capture individual-level health-related social needs, such as food insecurity, lack of transportation to frequent treatment appointments, and financial toxicity [6].

However, multilevel modeling suggests that while individual socioeconomic disadvantage drives a substantial portion of the observed disparities in cancer, neighborhood deprivation exerts a persistent, independent effect, even after adjusting for individual-level factors [7, 8]. A significant portion of this neighborhood-level effect is likely mediated by disparities in healthcare access, such as barriers to reaching specialized treatment centers. Furthermore, as clinical databases and cancer registries often lack comprehensive data on SDOH, residual confounding likely accounts for a portion of the variance attributed to neighborhood deprivation.

Therefore, the current operationalization of SDOH in oncological research presents significant limitations, resulting in a fragmented and incomplete understanding of a patient’s social condition. Addressing these measurement limitations will likely require health disparities researchers to employ community-engaged approaches that capture the nuanced, lived experiences of marginalized populations, rather than relying solely on retrospective data [9].

## Environmental Exposures Linked to the Development and Clinical Phenotype of Leukemia

Foundational studies on environmental exposures contributing to leukemia risk in the mid-20th century were based on occupational exposure to benzene and later radiation-induced leukemia among survivors of the atomic bombings in Hiroshima and Nagasaki. Several other leukemogenic environmental exposures have since been identified. One of the potential mechanisms through which SDOH can influence clinical phenotype of leukemia is through the inequitable distribution of environmental exposures. While prior chemotherapy, radiation therapy, and radiation exposure are well characterized leukemogenic risk factors [10–12], there is a growing body of evidence that chemical, occupational, and pollutant exposures can influence both the predisposition to develop acute leukemia and its biologic characteristics [13–16]. Recent studies have demonstrated a potential impact of air pollution, specifically exposure to polycyclic aromatic hydrocarbons and polycyclic organic matter (PAHPOM) on molecular characteristics of myeloid leukemias [17]. Similarly, within a cohort of patients with myelodysplastic syndrome (MDS), particulate matter 2.5 (PM2.5) exposure was associated with adverse genomic characteristics [18]. Air pollutant exposure and its impact on outcomes in ALL is also under investigation. In a California cohort of patients, neighborhood level pollution was associated with inferior outcomes and ozone exposure and PM2.5 were specifically associated with high-risk cytogenetic subtypes of ALL [19]. Current evidence suggests that vulnerable populations with acute leukemia are at higher risk of exposure to air pollutants, due to historic redlining practices within cities [20, 21], and these exposures are associated with adverse biologic characteristics of leukemia.

While food environment has not been directly studied, there are reported associations of obesity and leukemia incidence and outcomes. Maternal obesity has been linked to increased incidence of pediatric ALL [22], and in AML obesity is associated with intermediate-high risk cytogenetics [23]. Early preclinical studies have causally linked dietary fat to the expansion of premalignant clonal hematopoietic stem cells [24]. Elevated BMI predicted inferior non-relapse mortality and OS in ALL [25], but not AML which suggests differential toxicity of the treatment regimens utilized. While the above data are thought-provoking, further work to disentangle the direct impact of environmental exposures on disease biology versus the confounding of environmental exposures with receipt of suboptimal care is needed.

## Generalizability of Leukemia Risk Models

An emerging area of concern is the clinical relevance of the widely used European Leukemia Net (ELN) risk classification in diverse patient populations as these models were derived largely from AML patients of European ancestry with similar access to healthcare. Recent analyses from leukemia co-operative groups suggest that the favorable outcome typically conferred by NPM*1* mutations was not demonstrated in Black patients. Treatment of these patients on a clinical trial obviates differences related to access to care and suggests biologic differences based on SDOH or genomic ancestry which are difficult to dissect [26, 27]. A subsequent study mapped the national Area Deprivation Index onto ELN 2022 risk, showing that deprivation selectively shortened survival within the intermediate-risk group receiving intensive chemotherapy, whereas in favorable and adverse-risk disease, biology appeared to override a place-based SDOH signal [28]. Similarly loss of the favorable impact of *NOTCH1* mutations in Black patients with T-ALL may also suggest a role for SDOH in attenuating the benefit of favorable risk mutations and variable applicability of ALL risk scores when applied to a diverse population [29, 30]. Risk stratification has a significant downstream impact on treatment recommendations and decisions that are made with regards to intensity of therapy, consideration for clinical trials, and need for allogeneic stem cell transplant during consolidation. Ensuring the validity of current prognostic models in diverse populations needs to be a research priority given the downstream implications for care delivery.

However, understanding when outcomes are impacted by limited applicability of current risk models versus when they are driven by reduced access to care (e.g. access to specialty cancer centers, treatment delivery, clinical trial participation, and transplantation) will be an essential step in determining when our stratification tools are most useful.

## Diagnosis/Early Treatment

The management of acute leukemia is complex, requiring multidisciplinary expertise, rapid precision diagnostics, and, often, care at a specialty cancer center (SCC). Patients therefore benefit from timely, accessible, and comprehensive frontline care. Social determinants of health (SDOH) operate across every phase of this trajectory, beginning before a patient enters the cancer care system, influencing where care is sought, and affecting early treatment complications.

Area-level deprivation measures, including lower socioeconomic status (SES), lower median income, and population density, have been shown to predict reduced survival in ALL [19, 31, 32]. In AML, structural racism, operationalized as census-tract measures of disadvantage, affluence, and segregation, was shown to mediate much of the survival disparity between Non-Hispanic Black (NHB) and Hispanic patients compared with Non-Hispanic White (NHW) patients [33]. Additionally, residence in historically redlined neighborhoods is independently associated with greater mortality among children with cancer [34]. Together, these findings established that disparities in leukemia are rooted in the structural and economic conditions in which patients live, with consequences that extend into the early treatment period.

Frontline treatment setting is a key determinant of outcomes in acute leukemia. Treatment at SCCs confers a survival benefit, likely reflecting greater capacity to deliver risk-adapted chemotherapy, supportive care, and access to advanced therapies and clinical trials [35, 36]. This is especially relevant for adolescents and young adults (AYAs), who are more likely to receive pediatric-inspired ALL regimens at SCCs, translating into better outcomes [37, 38]. Accordingly, AYA ALL guidelines emphasize specialized, age-appropriate care [39, 40].

Access to SCCs is socially patterned by insurance, SES, language, transportation, caregiving demands, and travel distance. These barriers can delay referral and contribute to racial disparities in SCC access [41, 42]. Qualitative analyses investigating factors that contribute to increased time to diagnosis show that structural factors are a major domain of concern for patients [43].

In the induction period, social disadvantage manifests as higher acuity at presentation, greater early mortality, and unequal access to precision diagnostics. Children in lower SES neighborhoods and NHB children are more likely to require ICU admission at diagnosis [44, 45]. Similarly, race/ethnicity and neighborhood disadvantage have been shown to predict ICU admission during induction therapy for AML in adults, resulting in higher 90-day mortality and reduced long-term survival [46]. Public insurance further amplifies these disparities, with higher mortality during induction and a larger NHB-NHW survival gap observed among publicly insured patients [45]. Early mortality in AML is associated with single marital status, low neighborhood SES, and lack of insurance [47].

Molecular profiling at diagnosis and measurable residual disease following induction therapy now guide major treatment decisions in acute leukemia, including incorporation of targeted therapies that can deepen responses. In a single-institution AML analysis, Medicare-insured and underinsured patients were less likely to undergo next-generation sequencing, received less intensive therapy, and had inferior survival when compared to privately insured patients [48]. Treatment setting and access to care are crucial pathways through which SDOH influence leukemia care and outcomes.

## Access to Allogeneic Stem Cell Transplantation

Allogeneic stem cell transplantation offers the only potentially curative option available for advanced hematologic malignancies and is the most widely accepted path to a treatment-free remission in most cases of AML and a subset of ALL cases. These patients generally require treatment at specialized centers, which may require patients to obtain a referral and travel a distance – often over a long period of time and multiple visits, posing financial and psychosocial hardship.

Donor availability is inequitable, with underrepresentation of non-Caucasian ancestries in the National Marrow Donor Program (NMDP) registry limiting chances of finding a matched donor for minorities [49]. Additionally, attrition of donors from minorities is high, with reasons cited including ambivalence, lack of support, mistrust, religion and concerns about missing work/school/obligations [50]. Donor availability gaps have narrowed in recent years with improved outcomes using alternative unmatched donor sources, including cord blood with expansion techniques, mismatched unrelated donors, and haploidentical donors. Despite expanding non-HLA matched donor options, however, differences still exist. These include access, utilization and post-transplant outcomes. Utilization patterns in the US indicate that minorities, including Hispanics, non-Hispanic Blacks, and Asians, have lower rates of transplant than non-Hispanic Whites in acute leukemia [51–53].

SDOH measures that have been linked to lower likelihood of transplant include supplemental security income (SSI), lower income, lower education, never married status, and a lower neighborhood socioeconomic status index [54, 55]. Interestingly, Asians have lower transplant utilization rates despite higher median income, possibly related to other domains of SDOH such as language barriers and mistrust.

Inequalities also persist following transplant. Severe (grade 3–4) acute graft versus host disease (GVHD) was higher in non-Hispanic Black patients in a large CIBMTR study negatively impacting survival and transplant related mortality (TRM) [56]. Even after controlling for GVHD, household income was associated with TRM, relapse risk and survival. SDOH associated with increased risk of chronic GVHD included SSI and lack of telephone access. Post-transplant complications not only require additional resources from patients and caregivers, but in many cases lead to clinical decline, relapse, and death.

This highlights the multidimensional and complex nature of transplant disparities. The intricacy of cell therapies, combined with variable measures of SDOH, disease heterogeneity, treatments received and transplant center practices, results in varying findings in the literature. A recent​ study​, demonstrated no racial difference in outcomes after transplant in those surviving the first year, suggesting that transplant has the potential to level the field when access and early post-transplant complications are addressed [57]. There is no question that cell therapy programs need to better identify barriers relevant to their catchment area, to optimize access and utilization, as well as improve outcomes. In 2022, the American Society of Transplantation and Cellular Therapy (ASTCT) and the National Marrow Donor Program (NMDP) formed the ACCESS Initiative [58], exemplifying efforts needed to address persistent transplant disparities.

## Clinical Trial Inequities

Participation disparities in leukemia clinical trials are increasingly recognized as a structural access problem that threatens the generalizability of clinical research [42, 59–61]. Clinical trials provide access to novel therapies and serve as platforms for translational research and biobanking studies. Thus, participation that is not representative of the population with leukemia shapes both individual treatment opportunities as well as the scientific evidence generated for future patients. In acute leukemia, these disparities have been documented by race/ethnicity, age, sex, and other SDOH that may be proximal mediators of these larger demographic categorizations. These problems arise through issues at multiple points in the trial access pathway, including trial site availability, referral, eligibility, consent, logistical support, and retention. Much of the literature specific to leukemia has evaluated disparities by racial and ethnic categories. Retrospective analyses have shown that Hispanic patients and other non-White patients, including Black and Asian patients, are less likely to participate in therapeutic leukemia trials and linked biobank studies [42, 59, 60]. Importantly, these differences should not be interpreted as biologic or individual-level differences by race or ethnicity, but rather as demonstrations of structural barriers that affect whether patients can access, enroll in, and remain on a trial. In acute leukemia, these barriers may include differential access to trial sites, greater travel distance, lack of commercial insurance, limited English proficiency, competing financial demands, and caregiver availability [42, 59]. Even among patients with similar access to Comprehensive Cancer Centers, racial and ethnic disparities in enrollment may persist, suggesting that physical access to a trial site is necessary but not sufficient to attain representative participation [42].

A useful framework is to distinguish (1) trial availability, (2) eligibility, and (3) enrollment. Availability refers to whether a patient receives care at a site with a leukemia trial appropriate for their disease and place in their treatment journey. Eligibility reflects whether the patient meets a trial’s eligibility criteria, which may exclude individuals with comorbidities, organ dysfunction, poor performance status, or other clinical factors that are more common in medically underserved populations [62, 63]. Enrollment then depends on whether the trial is offered, whether the patient can complete consent, and whether practical supports are available for trial-related burdens including more frequent visits, testing, and potentially treatment-related toxicity. This distinction is important because analyses and interventions that address only one step, such as increasing site referral, may not reduce the overall disparity if strict eligibility criteria, language barriers, or lack of logistical support continue to prevent enrollment.

While not leukemia-specific, broader oncology literature supports findings that SDOH influence participation and may be a proximate cause of the racial and ethnic disparities discussed above. Lower socioeconomic affluence has been associated with decreased cancer trial enrollment, and limited English proficiency has been linked to reduced participation among culturally and linguistically diverse patients [64, 65]. Insurance status may also shape access, not necessarily through insurance status alone, but through variable coverage of trial-related costs, out-of-network care, travel, missed work, and ancillary expenses [66, 67]. These barriers can compound existing inequities when they intersect with structural racism, residential segregation, lower access to specialty centers, and differences in healthcare resources.

Future work should move beyond documenting disparities toward identifying where in the trial pathway patients are lost, what the proximal reasons were, and testing solutions to overcome those barriers. Prospective studies should measure trial availability, referral patterns, offer rates, reasons for ineligibility, reasons for refusal, language needs, caregiver availability, transportation barriers, financial burden, and retention after enrollment. Interventions should be based on and matched to specific barriers and utilize established intervention structures such as trial navigation. At the structural level, modernizing eligibility criteria may improve inclusion of patients who have historically been excluded because of comorbidities or laboratory thresholds that are not essential for safety [62, 63]. Other strategies may include embedding trials at community sites, streamlining referral pathways to leukemia centers, providing translated consent materials and interpreter services, reimbursing transportation and lodging, supporting caregivers, and partnering with trusted community organizations [68]. Together, these approaches would help ensure that leukemia trials are more accessible, more representative, and more relevant to the populations affected by the disease.

## Disparities in Utilization of Palliative Care

Patients with acute leukemia experience substantial physical and psychological symptom burden related to both disease and treatment, yet palliative care remains underutilized in this population [69–71]. Despite evidence that early integrated palliative care improves quality of life, psychological outcomes, and end-of-life care in acute myeloid leukemia (AML), only 14% of AML hospitalizations include palliative care encounters [69, 72]. Within the broader care continuum, social determinants of health shape not only access to palliative care, but also the intensity, location, and cost of end-of-life care.

Racial and ethnic disparities in palliative care access are persistent and profound. Patients of Hispanic ethnicity with AML have significantly lower odds of receiving palliative care when compared with non-Hispanic White patients (OR 0.73; 95% CI 0.59–0.90) [70]. Non-Hispanic Black patients with hematologic malignancies are less likely to receive palliative care consultation or complete advance directives. They are thus, more likely to experience intensive end-of-life care, including ICU utilization and in-hospital death [72]. These patterns reflect structural inequities in care delivery. Measures of structural racism, including census-level disadvantage and segregation indices, account for a substantial proportion of survival disparities between non-Hispanic Black and non-Hispanic White patients with AML, operating through differential treatment patterns and access to care [33]. Patients residing in predominantly non-Hispanic Black neighborhoods face a greater than 20% increased risk of death compared to those in predominantly non-Hispanic White neighborhoods [73].

Socioeconomic context profoundly influences both access to palliative services and patterns of end-of-life care utilization. Patients in the most deprived neighborhoods are more likely to die in-hospital versus at home (OR 1.30), receive acute hospital-based care in the last three months of life (OR 1.16), and not receive specialist palliative care (OR 1.13) [74]. Increasing neighborhood deprivation is associated with progressively lower access to palliative care [74]. Low socioeconomic status is also linked to reduced hospice utilization, with an estimated 18% decrease [75]. Insurance status further shapes care delivery and outcomes, as patients with Medicaid, Medicare, or are uninsured have a 17–21% higher mortality risk compared to those with private insurance [74]. Notably, Medicaid expansion has been associated with increased palliative care utilization [76].

Transfusion dependence creates a unique structural and economic barrier for patients with acute leukemia. Patients requiring ongoing transfusions have 31% lower odds of enrolling in hospice and experience markedly shorter hospice stays (median 6 versus 11 days) [77]. Approximately two-thirds of patients who disenroll from hospice do so to receive transfusions, underscoring its central role in decision-making [77]. Current reimbursement structures reinforce this barrier, as the fixed Medicare hospice per diem rate ($224.62 in 2025) limits most hospice agencies, particularly those serving under-resourced communities, from offering palliative transfusions, creating a payment-driven barrier that disproportionately affects vulnerable populations [71, 77].

Reducing these disparities will require multilevel interventions that target both care delivery and payment structures. Automatic palliative care consultation triggers at diagnosis may reduce referral variability [69, 78]. Culturally concordant communication and professional interpretation can bridge gaps in health literacy and trust [79]. Payment reform to support palliative transfusions in hospice could address a key structural barrier and shift care toward lower-intensity settings [71, 77]. Expanding community-based palliative care, particularly in disenfranchised infrastructures, is critical [77, 80]. Addressing upstream social determinants through housing stability, transportation assistance, and financial support may reduce cumulative disadvantage [81, 82].

## Conclusion

**Fig. 1:**
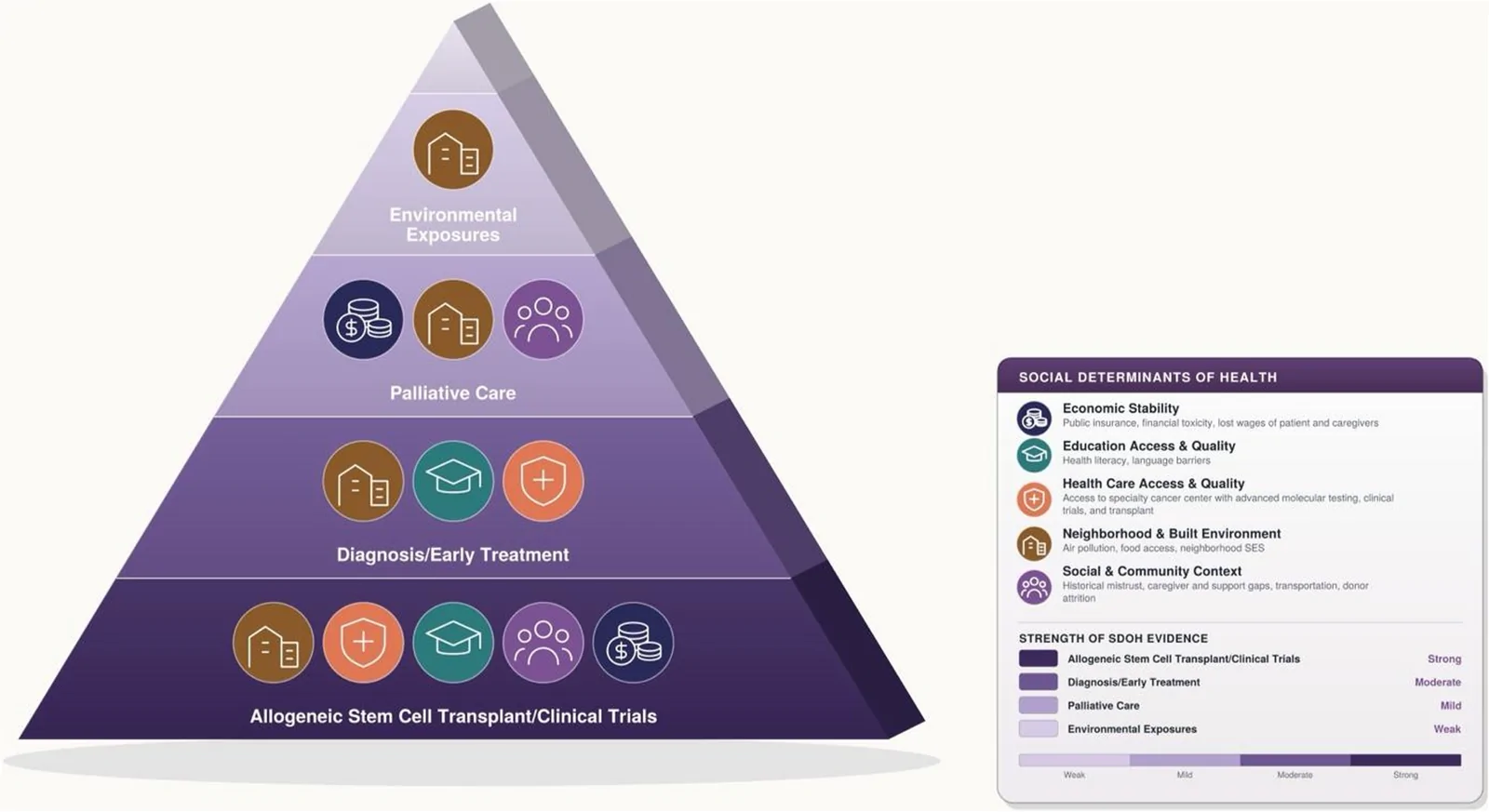
A tiered framework illustrating the domain-specific influence of social determinants of health across the acute leukemia care continuum, with domain icons indicating specific determinants acting at each tier and associated pressures detailed in the legend. Tiers are ordered and shaded by strength of evidence​. This figure was designed by the authors and rendered with the assistance of a large language model (Claude, Anthropic); all content and interpretations belong to the authors

Current evidence suggests that SDOH significantly underpin well-established racial disparities in leukemia outcomes. Social determinants of health form an interwoven meshwork with the acute leukemia patient care continuum from disease development to diagnosis and treatment, including end-of-life care (Fig. 1). However, current data utilize heterogeneous measures of neighborhood SES without incorporating individual-level SDOH measures. Future efforts are needed to develop comprehensive SDOH measures and integrate them into the diagnostic workflow of acute leukemia patients where they can complement genomic data to guide clinical decision making. In addition, community-academic partnerships must be strengthened to improve access to specialized care including clinical trials, allogeneic stem cell transplant and palliative care. These multi-level efforts will represent key steps toward achieving more equitable outcomes for patients with acute leukemia.

## Key References

- Abraham IE, Rauscher GH, Patel AA, Pearse WB, Rajakumar P, Burkart M, et al. Structural racism is a mediator of disparities in acute myeloid leukemia outcomes. Blood. 2022;139:2212–26.
  - ○ An analysis of a real-world cohort of adult patients with AML that established neighborhood SES is a potent mediator of racial disparities in AML survival.
- Wolfson J, Sun C-L, Wyatt L, Stock W, Bhatia S. Adolescents and Young Adults with Acute Lymphoblastic Leukemia and Acute Myeloid Leukemia: Impact of Care at Specialized Cancer Centers on Survival Outcome. Cancer Epidemiol Biomarkers Prev. 2017;26:312–20.
  - ○ This study demonstrated that AYAs with acute leukemia treated at specialized cancer centers have superior survival, establishing treatment setting as a key determinant of outcomes.
- Ashok Kumar P, Ghimire K, Haroun E, et al. Utilization and outcome disparities in allogeneic hematopoietic stem cell transplant in the United States. Eur J Haematol. 2024;112(3):328–338.
  - ○ This comprehensive review of the US transplant system covers disparities due to race/ethnicity, socioeconomic status, and social determinants of health and how they may impact access, utilization and outcomes. It also reviews effort in place to address these and shortcomings, highlighting ongoing needs.
- Hantel A, Wang Y, Cronin A, Khan I, Abraham I, Eisfeld A-K, et al. Impact of modernizing eligibility criteria on enrollment and representation in acute myeloid leukemia clinical trials. Blood. 2026;147:639–49.
  - ○ In this analysis of a retrospective cohort of 2226 newly diagnosed patients across 8 hospitals, modernization of eligibility for AML trials to focus on safety-based criteria was shown to improve both overall enrollment and population representation.
- El-Jawahri A, Nelson AM, Gray TF, Lee SJ, LeBlanc TW. Palliative and End-of-Life Care for Patients With Hematologic Malignancies. J Clin Oncol. 2020 Mar 20;38(9):944–953.
  - ○ This comprehensive review provides a foundational overview of the unique barriers to palliative care integration within hematologic malignancies, including prognostic uncertainty, treatment intensity near end of life, and low hospice utilization.

## Data Availability

No datasets were generated or analysed during the current study.

## References

[1] Social determinants of health [Internet]. [cited 2026 June 30]. https://www.who.int/health-topics/social-determinants-of-health. Accessed 30 June 2026.

[2] Sharara M, Tjioe KC, Miranda-Galvis M, Santellano Juarez B, Agrawal G, Balas EA, et al. Social determinants of health impact on cancer affecting children, adolescents, and young adults: systematic review and meta-analysis. Front Adolesc Med. 2024;2:1441776. 10.3389/fradm.2024.1441776.

[3] Oakes JM, Rossi PH. The measurement of SES in health research: current practice and steps toward a new approach. Soc Sci Med. 2003;56:769–84. 10.1016/S0277-9536(02)00073-4. 12560010

[4] Yu Z, Yang X, Guo Y, Bian J, Wu Y. Assessing the documentation of social determinants of health for lung cancer patients in clinical narratives. Front Public Health. 2022;10:778463. 10.3389/fpubh.2022.778463. PMC8995779 35419333

[5] Flanagan BE, Gregory EW, Hallisey EJ, Heitgerd JL, Lewis B. A social vulnerability index for disaster management. J Homeland Secur Emerg Manag. 2011;8:0000102202154773551792. 10.2202/1547-7355.1792.

[6] Miranda-Galvis M, Tjioe KC, Balas EA, Agrawal G, Cortes JE. Disparities in survival of hematologic malignancies in the context of social determinants of health: a systematic review. Blood Adv. 2023;7:6466–91. 10.1182/bloodadvances.2023010690. PMC10632659 37639318

[7] Gomez SL, Shariff-Marco S, DeRouen M, Keegan THM, Yen IH, Mujahid M, et al. The impact of neighborhood social and built environment factors across the cancer continuum: current research, methodological considerations, and future directions. Cancer. 2015;121:2314–30. 10.1002/cncr.29345. PMC4490083 25847484

[8] Sorice KA, Fang CY, Wiese D, Ortiz A, Chen Y, Henry KA, et al. Systematic review of neighborhood socioeconomic indices studied across the cancer control continuum. Cancer Med. 2022;11:2125–44. 10.1002/cam4.4601. PMC9119356 35166051

[9] Ford CL, Airhihenbuwa CO. Critical Race Theory, Race Equity, and Public Health: toward antiracism praxis. Am J Public Health. 2010;100:S30-5. 10.2105/AJPH.2009.171058. PMC2837428 20147679

[10] Singhal D, Kutyna MM, Hahn CN, Shah MV, Hiwase DK. Therapy-related myeloid neoplasms: complex interactions among cytotoxic therapies, genetic factors, and aberrant microenvironment. Blood Cancer Discov. 2024;5:400–16. 10.1158/2643-3230.BCD-24-0103. PMC11528189 39422544

[11] Bosch De Basea M, Thierry-Chef I, Harbron R, Hauptmann M, Byrnes G, Bernier M-O, et al. Risk of hematological malignancies from CT radiation exposure in children, adolescents and young adults. Nat Med. 2023;29:3111–9. 10.1038/s41591-023-02620-0. PMC10719096 37946058

[12] Patel AA, Rojek AE, Drazer MW, Weiner H, Godley LA, Le Beau MM, et al. Therapy-related myeloid neoplasms in 109 patients following radiation monotherapy. Blood Adv. 2021:bloodadvances.2021004964. 10.1182/bloodadvances.2021004964. PMC8945635 34492705

[13] Jasra S, Giricz O, Zeig-Owens R, Pradhan K, Goldfarb DG, Barreto-Galvez A, et al. High burden of clonal hematopoiesis in first responders exposed to the World Trade Center disaster. Nat Med. 2022;28:468–71. 10.1038/s41591-022-01708-3. PMC9394171 35256801

[14] Poynter JN, Richardson M, Roesler M, Blair CK, Hirsch B, Nguyen P, et al. Chemical exposures and risk of acute myeloid leukemia and myelodysplastic syndromes in a population-based study. Intl J Cancer. 2017;140:23–33. 10.1002/ijc.30420. PMC5245124 27603749

[15] Gilbert J, Abbott D, Molina Kuna E, Perraillon M, Patel A, Pollyea D. County-level analysis reveals two high-incidence regions and potential environmental contributors to myeloid malignancies in Colorado. Blood. 2025;146:2853–2853. 10.1182/blood-2025-2853.

[16] Lin C-K, Hsu Y-T, Brown KD, Pokharel B, Wei Y, Chen S-T. Residential exposure to petrochemical industrial complexes and the risk of leukemia: a systematic review and exposure-response meta-analysis. Environ Pollut. 2020;258:113476. 10.1016/j.envpol.2019.113476. 31902537

[17] Palmer A, Rauscher G, Abraham I, Burkart M, Dave A, Raptis N, et al. Air pollutant impact on disease characteristics and outcomes in patients with acute myeloid leukemia. Blood Adv. 2024;8:4647–50. 10.1182/bloodadvances.2024013280. PMC11402143 38810264

[18] Verma M, Davidsohn MP, Arends CM, Verma D, Sahu S, Pradhan K, et al. Environmental pollution triggers inflammation in vivo and is associated with higher risk MDS in an urban cohort. Am J Hematol. 2026;101:346–9. 10.1002/ajh.70132. 41215735

[19] Hernandez F, Stewart E, Morimoto LM, Kang AY, Winestone LE, Metayer C. The impact of environmental contaminants, air pollution, and social deprivation on childhood leukemia survival in California. Front Oncol. 2025;15:1686115. 10.3389/fonc.2025.1686115. PMC12719266 41438983

[20] Tessum CW, Paolella DA, Chambliss SE, Apte JS, Hill JD, Marshall JD. PM2.5 polluters disproportionately and systemically affect people of color in the United States. Sci Adv. 2021;7:eabf4491. 10.1126/sciadv.abf4491. PMC11426197 33910895

[21] Lane HM, Morello-Frosch R, Marshall JD, Apte JS. Historical redlining is associated with present-day air pollution disparities in U.S. cities. Environ Sci Technol Lett. 2022;9:345–50. 10.1021/acs.estlett.1c01012. PMC9009174 35434171

[22] Liu J, Kharazmi E, Liang Q, Chen Y, Sundquist J, Sundquist K, et al. Maternal weight during pregnancy and risk of childhood acute lymphoblastic leukemia in offspring. Leukemia. 2025;39:590–8. 10.1038/s41375-025-02517-6. PMC11879861 39865137

[23] Finn L, Sproat L, Heckman MG, Jiang L, Diehl NN, Ketterling R, et al. Epidemiology of adult acute myeloid leukemia: impact of exposures on clinical phenotypes and outcomes after therapy. Cancer Epidemiol. 2015;39:1084–92. 10.1016/j.canep.2015.09.003. 26365691

[24] Pasupuleti SK, Ramdas B, Burns SS, Palam LR, Kanumuri R, Kumar R, et al. Obesity-induced inflammation exacerbates clonal hematopoiesis. J Clin Invest. 2023;133:e163968. 10.1172/JCI163968. PMC10231999 37071471

[25] Shimony S, Flamand Y, Valtis YK, Place AE, Silverman LB, Vrooman LM, et al. Effect of BMI on toxicities and survival among adolescents and young adults treated on DFCI Consortium ALL trials. Blood Adv. 2023;7:5234–45. 10.1182/bloodadvances.2023009976. PMC10500474 37432068

[26] Bhatnagar B, Kohlschmidt J, Mrózek K, Zhao Q, Fisher JL, Nicolet D, et al. Poor survival and differential impact of genetic features of Black patients with acute myeloid leukemia. Cancer Discov. 2021;11:626–37. 10.1158/2159-8290.CD-20-1579. PMC7933110 33277314

[27] Stiff A, Fornerod M, Kain BN, Nicolet D, Kelly BJ, Miller KE, et al. Multiomic profiling identifies predictors of survival in African American patients with acute myeloid leukemia. Nat Genet. 2024;56:2434–46. 10.1038/s41588-024-01929-x. PMC11549055 39367245

[28] Shimony S, Wang Y, Cronin A, Walsh TP, Charles A, Slopen N, et al. Stratifying survival: the role of social determinants of health on AML outcomes by ELN 2022 criteria. Blood Adv. 2022;10:1879–82. 10.1182/bloodadvances.2025018032. PMC12993897 41538295

[29] Newman H, Lee SHR, Pölönen P, Shraim R, Li Y, Liu H, et al. Impact of genetic ancestry on genomics and survival outcomes in T-cell acute lymphoblastic leukemia. Blood Cancer Discov. 2025;6:412–24. 10.1158/2643-3230.BCD-25-0049. PMC12405861 40434808

[30] Simonin M, Vasseur L, Lengliné E, Lhermitte L, Cabannes-Hamy A, Balsat M, et al. NGS-based stratification refines the risk stratification in T-ALL and identifies a very-high-risk subgroup of patients. Blood. 2024;144:1570–80. 10.1182/blood.2023023754. 38848537

[31] Hoppmann AL, Hurley DM, Cramer S, Brown MJ, Ghosal R, Alberg AJ, et al. Neighborhood socioeconomic status and overall survival among children with acute lymphoblastic leukemia. Blood Adv. 2025;9:5861–9. 10.1182/bloodadvances.2025017044. PMC12663511 40845260

[32] Sasaki K, Jabbour E, Short NJ, Jain N, Ravandi F, Pui C, et al. Acute lymphoblastic leukemia: A population-based study of outcome in the U nited S tates based on the surveillance, epidemiology, and end results (SEER) database, 1980–2017. Am J Hematol. 2021;96:650–8. 10.1002/ajh.26156. PMC9517941 33709456

[33] Abraham IE, Rauscher GH, Patel AA, Pearse WB, Rajakumar P, Burkart M, et al. Structural racism is a mediator of disparities in acute myeloid leukemia outcomes. Blood. 2022;139:2212–26. 10.1182/blood.2021012830. PMC9710198 35061876

[34] Karvonen KA, Vu A, Lin K, Gibbons J, Mendoza JA, Chow EJ, et al. Historical redlining and mortality in children, adolescents, and young adults with cancer in California, 2000–2019. JNCI J Natl Cancer Inst. 2000;117:1646–54. 10.1093/jnci/djaf105. PMC12756997 40272942

[35] Bhatt VR, Shostrom V, Giri S, Gundabolu K, Monirul Islam KM, Appelbaum FR, et al. Early mortality and overall survival of acute myeloid leukemia based on facility type. Am J Hematol. 2017;92:764–71. 10.1002/ajh.24767. 28437868

[36] Khullar K, Plascak JJ, Parikh RR. Acute lymphoblastic leukemia (ALL) in adults: disparities in treatment intervention based on access to treatment facility. Leuk Lymphoma. 2022;63:170–8. 10.1080/10428194.2021.1975187. 34493143

[37] Muffly LS, Parsons HM, Miller K, Li Q, Brunson A, Keegan TH. Impact of specialized treatment setting on survival in adolescent and young adult ALL. JCO Oncol Pract. 2023;19:1190–8. 10.1200/OP.23.00373. PMC12777624 37890123

[38] Wolfson J, Sun C-L, Wyatt L, Stock W, Bhatia S. Adolescents and Young Adults with Acute Lymphoblastic Leukemia and Acute Myeloid Leukemia: Impact of Care at Specialized Cancer Centers on Survival Outcome. Cancer Epidemiol Biomarkers Prev. 2017;26:312–20. 10.1158/1055-9965.EPI-16-0722. PMC5546404 28209594

[39] Bhatia S, Pappo AS, Acquazzino M, Allen-Rhoades WA, Barnett M, Borinstein SC, et al. Adolescent and Young Adult (AYA) Oncology, Version 2.2024, NCCN Clinical Practice Guidelines in Oncology. J Natl Compr Canc Netw. 2023;21:851–80. 10.6004/jnccn.2023.0040. 37549914

[40] DuVall AS, McNeer J, Cheung MC, Adrianzen-Herrera DA, Advani AS, Aljurf M, et al. ASH 2026 Guidelines for Frontline Management of Acute Lymphoblastic Leukemia in Adolescents and Young Adults. Blood Adv. 2026. 10.1182/bloodadvances.2021006469. bloodadvances.2021006469. PMC13366240 41670627

[41] Parsons HM, Muffly LS, Garcia A, Zhang A, Miller K, Van Riper D, et al. Travel-time barriers to specialized cancer care for adolescents and young adults with acute lymphoblastic leukemia. JNCI Cancer Spectr. 2024;8:pkae046. 10.1093/jncics/pkae046. PMC11229987 38845074

[42] Hantel A, Brunner AM, Plascak JJ, Uno H, Varela JC, Luskin MR, et al. Racial and ethnic associations with comprehensive cancer center access and clinical trial enrollment for acute leukemia. JNCI J Natl Cancer Inst. 2024;116:1178–84. 10.1093/jnci/djae067. PMC11223832 38518098

[43] Ding L, Szymczak JE, Evans E, Canepa E, Martin AE, Contractor F, et al. Factors that contribute to disparities in time to acute leukemia diagnosis in young people: an in depth qualitative interview study. BMC Cancer. 2022;22:531. 10.1186/s12885-022-09547-8. PMC9095817 35550034

[44] Swift CM, Chism E, Lin K, Cortella A, Banerjee T, Alvarez JS, et al. Lower neighborhood socioeconomic status is associated with intensive care unit admission at initial presentation in children with acute leukemia. Pediatr Blood Cancer. 2025;72:e32049. 10.1002/pbc.32049. 40947565

[45] Winestone LE, Getz KD, Miller TP, Wilkes JJ, Sack L, Li Y, et al. The role of acuity of illness at presentation in early mortality in black children with acute myeloid leukemia. Am J Hematol. 2017;92:141–8. 10.1002/ajh.24605. PMC5733783 27862214

[46] Abraham IE, Rauscher GH, Luo J, Monick S, Burkart M, Doukas P, et al. Clinical and sociodemographic predictors of intensive care unit admission following chemotherapy in acute myeloid leukemia. Haematologica. 2026. 10.3324/haematol.2025.288876. PMC13317841 41537345

[47] Ho G, Jonas BA, Li Q, Brunson A, Wun T, Keegan THM. Early mortality and complications in hospitalized adult Californians with acute myeloid leukaemia. Br J Haematol. 2017;177:791–9. 10.1111/bjh.14631. PMC5444943 28419422

[48] Bade R, Banaszak LG, Osman F, Cabral P, Hassan A, Stepanovic A, et al. Neighborhood disadvantage, insurance status, and molecular profiling of patients with acute myeloid leukemia. Leuk Res. 2023;131:107326. 10.1016/j.leukres.2023.107326. 37263074

[49] Dehn J, Arora M, Spellman S, Setterholm M, Horowitz M, Confer D, et al. Unrelated donor hematopoietic cell transplantation: factors associated with a better HLA match. Biol Blood Marrow Transplant. 2008;14:1334–40. 10.1016/j.bbmt.2008.09.009. PMC3319684 19041054

[50] Fingrut WB, Gyurkocza B, Davis E, Flynn J, Chinapen S, Naputo KA, et al. Racial disparities in access to alternative donor allografts persist in the era of donors for all. Blood Adv. 2022;6:5625–9. 10.1182/bloodadvances.2022007814. PMC9642869 35675518

[51] Ashok Kumar P, Ghimire K, Haroun E, Kassab J, Saba L, Gentile T, et al. Utilization and outcome disparities in allogeneic hematopoietic stem cell transplant in the United States. Eur J Haematol. 2024;112:328–38. 10.1111/ejh.14129. 37899652

[52] Kang LC, Fiala MA, Johnson KJ. Disparities in allogeneic hematopoietic stem cell transplantation among acute myeloid leukemia patients. Transplant Cell Ther. 2026;32:723–34. 10.1016/j.jtct.2026.01.019. 41547398

[53] Landry I. Racial disparities in hematopoietic stem cell transplant: a systematic review of the literature. Stem Cell Investig. 2021;8:24. 10.21037/sci-2021-058. PMC8743384 35071585

[54] Jabo B, Morgan JW, Martinez ME, Ghamsary M, Wieduwilt MJ. Sociodemographic disparities in chemotherapy and hematopoietic cell transplantation utilization among adult acute lymphoblastic and acute myeloid leukemia patients. PLoS One. 2017;12:e0174760. 10.1371/journal.pone.0174760. PMC5383052 28384176

[55] Wuliji N, Jones SMW, Gooley T, Gerds AT, Medeiros BC, Shami PJ, et al. Social determinants of health and access to allogeneic hematopoietic cell transplantation for acute myeloid leukemia. Blood. 2025;145:3041–51. 10.1182/blood.2024027543. PMC13122322 40067123

[56] Farhadfar N, Rashid N, Chen K, DeVos J, Wang T, Ballen K, et al. Racial, ethnic, and socioeconomic diversity and outcomes of patients with graft-versus-host disease: a CIBMTR analysis. Blood Adv. 2024;8:4963–76. 10.1182/bloodadvances.2024013074. PMC11496972 38776400

[57] Blue BJ, Brazauskas R, Chen K, Patel J, Zeidan AM, Steinberg A, et al. Racial and socioeconomic disparities in long-term outcomes in ≥ 1 year allogeneic hematopoietic cell transplantation survivors: a CIBMTR analysis. Transplant Cell Ther. 2023;29:709.e1-709.e11. 10.1016/j.jtct.2023.07.013. PMC11258715 37482244

[58] Auletta JJ, Sandmaier BM, Jensen E, Majhail NS, Knutson J, Nemecek E, et al. The ASTCT-NMDP ACCESS Initiative: A Collaboration to Address and Sustain Equal Outcomes for All across the Hematopoietic Cell Transplantation and Cellular Therapy Ecosystem. Transpl Cell Ther. 2022;28:802–9. 10.1016/j.jtct.2022.09.020. 36184058

[59] Hantel A, Kohlschmidt J, Eisfeld A-K, Stock W, Jacobson S, Mandrekar S, et al. Inequities in Alliance Acute Leukemia Clinical Trial and Biobank Participation: Defining Targets for Intervention. JCO. 2022;40:3709–18. 10.1200/JCO.22.00307. PMC9649272 35696629

[60] Loeb N, Katsnelson O, Jain A, Tahvildar P, Teitelbaum D, Garcia-Horton A. Race and age disparities in randomized trials of acute myeloid leukemia: a systematic review and meta-analysis. Blood. 2025;2:100070. 10.1016/j.bneo.2025.100070. PMC12067879 40453142

[61] Gong IY, Soto MJ, Rafinejad-Farahani B, Unger JM, Conti RM, Guerra CE, et al. Disparities in clinical trial participation among older adult Medicare beneficiaries with hematologic malignancies from 2006 to 2019: a SEER–Medicare analysis. Cancer. 2006;131:e70204. 10.1002/cncr.70204. PMC12690631 41368947

[62] Pulte D, Fernandes L, Wei G, Woods A, Norsworthy KJ, Gormley N, et al. FDA Analysis of Ineligibility for Acute Myeloid Leukemia Clinical Trials by Race and Ethnicity. Clin Lymphoma Myeloma Leuk. 2023;23:463–e4701. 10.1016/j.clml.2023.03.012. 37076368

[63] Hantel A, Wang Y, Cronin A, Khan I, Abraham I, Eisfeld A-K, et al. Impact of modernizing eligibility criteria on enrollment and representation in acute myeloid leukemia clinical trials. Blood. 2026;147:639–49. 10.1182/blood.2025030741. PMC13000933 41100728

[64] Donzo MW, Nguyen G, Nemeth JK, Owoc MS, Mady LJ, Chen AY, et al. Effects of socioeconomic status on enrollment in clinical trials for cancer: a systematic review. Cancer Med. 2024;13:e6905. 10.1002/cam4.6905. PMC10807561 38169154

[65] Smith A‘B, Agar M, Delaney G, Descallar J, Dobell-Brown K, Grand M, et al. Lower trial participation by culturally and linguistically diverse (CALD) cancer patients is largely due to language barriers. Asia-Pac J Clin Oncol. 2018;14:52–60. 10.1111/ajco.12818. 29083094

[66] Caston NE, Lalor F, Wall J, Sussell J, Patel SA, Williams C, et al. Associations between insurance status and the cancer clinical trial enrollment process. JCO. 2022;40:85–85. 10.1200/JCO.2022.40.28_suppl.085. PMC9653198 36178922

[67] Klamerus JF, Bruinooge SS, Ye X, Klamerus ML, Damron D, Lansey D, et al. The Impact of Insurance on Access to Cancer Clinical Trials at a Comprehensive Cancer Center. Clin Cancer Res. 2010;16:5997–6003. 10.1158/1078-0432.CCR-10-1451. PMC3715082 21169253

[68] Heller C, Balls-Berry JE, Nery JD, Erwin PJ, Littleton D, Kim M, et al. Strategies addressing barriers to clinical trial enrollment of underrepresented populations: a systematic review. Contemp Clin Trials. 2014;39:169–82. 10.1016/j.cct.2014.08.004. PMC6936726 25131812

[69] El-Jawahri A, Nelson AM, Gray TF, Lee SJ, LeBlanc TW. Palliative and end-of-life care for patients with hematologic malignancies. J Clin Oncol. 2020;38:944–53. 10.1200/JCO.18.02386. PMC8462532 32023164

[70] Sanders JJ, Temin S, Ghoshal A, Alesi ER, Ali ZV, Chauhan C, et al. Palliative care for patients with cancer: ASCO guideline update. J Clin Oncol. 2024;42:2336–57. 10.1200/JCO.24.00542. 38748941

[71] El-Jawahri A, LeBlanc TW, Kavanaugh A, Webb JA, Jackson VA, Campbell TC, et al. Effectiveness of Integrated Palliative and Oncology Care for Patients With Acute Myeloid Leukemia: A Randomized Clinical Trial. JAMA Oncol. 2021;7:238. 10.1001/jamaoncol.2020.6343. PMC7747042 33331857

[72] Amaeshi L, Imeh M. Temporal trends and determinants of palliative care utilization among hospitalized patients with acute myeloid leukemia. J Clin Oncol. 2025. 10.1200/JCO.2025.43.16_suppl.e13832. PMC12771721 41327169

[73] Thida AM, Erdinc B, Homsy S, Jafri FM, Chiu E, Gonsky JP. Health disparities in acute myeloid leukemia: a decade-long analysis of Afro-Caribbean patients in a safety net New York City hospital. J Clin Oncol. 2024;42:e18515. 10.1200/JCO.2024.42.16_suppl.e18515.

[74] Potenza L, Giusti D, Borelli E, Colaci E, Banchelli F, Cuoghi Costantini R, et al. Early Palliative Care, Goals of Care Conversations and Quality EOL Care in Acute Leukemia and HR-MDS. J Pain Symptom Manag. 2025;70:121–e1303. 10.1016/j.jpainsymman.2025.03.029. 40187378

[75] Fei-Zhang DJ, Wu E, Stanisic AV, Hou L, Platanias LC, Ansell SM, et al. Socioeconomic, racial-ethnic, household, and infrastructural disparities of hematologic cancer outcomes in the United States. Blood Adv. 2025;9:1463–71. 10.1182/bloodadvances.2024013956. PMC12094250 39808792

[76] LeBlanc TW, Egan PC, Olszewski AJ. Transfusion dependence, use of hospice services, and quality of end-of-life care in leukemia. Blood. 2018;132:717–26. 10.1182/blood-2018-03-842575. PMC6097134 29848484

[77] Raman HS, Cronin AM, Huntington SF, Uno H, Brennan C, Hurley SL, et al. Perceived value of transfusion access and hospice services among patients with blood cancers. JAMA Netw Open. 2025;8:e2541719. 10.1001/jamanetworkopen.2025.41719. PMC12590303 41191354

[78] Kirtane K, Downey L, Lee SJ, Curtis JR, Engelberg RA. Intensity of End-of-Life Care for Patients with Hematologic Malignancies and the Role of Race/Ethnicity. J Palliat Med. 2018;21:1466–71. 10.1089/jpm.2018.0152. PMC6201780 29975599

[79] Wang R, Zeidan AM, Halene S, Xu X, Davidoff AJ, Huntington SF, et al. Health care use by older adults with acute myeloid leukemia at the end of life. J Clin Oncol. 2017;35:3417–24. 10.1200/JCO.2017.72.7149. PMC5648174 28783450

[80] Davies JM, Sleeman KE, Leniz J, Wilson R, Higginson IJ, Verne J, et al. Socioeconomic position and use of healthcare in the last year of life: A systematic review and meta-analysis. Prigerson HG, editor. PLoS Med. 2019;16:e1002782. 10.1371/journal.pmed.1002782. PMC6478269 31013279

[81] Lai Y-J, Chen Y-Y, Ko M-C, Chou Y-S, Huang L-Y, Chen Y-T, et al. Low Socioeconomic Status Associated With Lower Utilization of Hospice Care Services During End-of-Life Treatment in Patients With Cancer: A Population-Based Cohort Study. J Pain Symptom Manag. 2020;60:309–e3151. 10.1016/j.jpainsymman.2020.03.015. 32240750

[82] Master S, Munker R, Shi Z, Mills G, Shi R. Insurance Status and Other Non-biological Factors Predict Outcomes in Acute Myelogenous Leukemia: Analysis of Data from the National Cancer Database. AR. 2016;36:4915–22. 10.21873/anticanres.11057. 27630349

